# Case of Poisoning by Opium Treated, with Belladonna

**Published:** 1856-03

**Authors:** Wm. H. Mussey


*Case of Poisoning by Opium treated with Belladonna.* Reported by WM. H. MUSSEY, M. D.

At midnight, October 29th, I visited a female, twenty-four years of age, who was said to have taken laudanum for the purpose of self-destruction. I found her comatose, with stertorous breathing, pulse feeble, 50 per minute. Surface cold, and pupils contracted to a mere speck. At 7 P. M., the patient had swallowed one ounce of the tincture of opium, in the presence of another person. A physician was summoned, who essayed to use the stomach pump, but so effectual was the resistance as to create the belief that no laudanum had been taken, and the doctor departed. Later, a disciple of Hahnemann administered of his *arca arcanorum*, so as not to offend the delicate stomach, but on a second visit, (three hours after the poison was taken,) his faith in pellets languished, and becoming heroic, he ordered a strong decoction of coffee, in such quantities as to produce vomiting. The patient was kept "quiet" with cold water to the head, and her friends assured her there was no danger. Two hours later, I was called, and finding the patient in the state where effort at resuscitation is usually considered useless, I determined to try the effect of Belladonna, as suggested by Dr. Thos. Anderson, and ordering extract of Belladonna, 8 grains, in 2 ounces water, I commenced giving by the teaspoonful; as the fluid accumulated in the mouth, it was necessary to raise the head to cause its passage to the stomach. Each successive act of deglutition was attended with increased difficulty till I feared to administer any more, lest the patient should strangle. *Seven grains* of the "*Extract*" were thus administered. Watching closely for a half hour, I observed the rigidity of the contraction of the pupil to relax slightly, but no other sign of improvement. At 1 o'clock, by means of a tube passed into the stomach, I injected *one ounce* of the *tincture* of Belladonna. At 2 o'clock the pupil had dilated to three times its former diameter, the pulse, respiration and temperature of the skin had improved. At 3 o'clock, the skin was warm, pulse 100 per minute, respiration easy, and the general appearance as of a quiet sleep, but as yet there was no sign of consciousness. Considering the symptoms entirely favorable to recovery, I left the patient. At 8 o'clock I called again. The patient had awakened at 6 o'clock, complained of not being able to see distinctly for a few hours, and could not stand upon her feet till evening. There was no preternatural dilation of the pupil, dryness of the fauces, heat or redness of the skin, resulting from the Belladonna (7 grains of the extract, and one ounce of the tincture) taken into the stomach.—*Cincinnati Med. Observer.*

